# Traumatic blunt thoracic aortic injury: a 10-year single-center retrospective analysis

**DOI:** 10.1186/s13019-022-02094-0

**Published:** 2022-12-23

**Authors:** Jingwei Sun, Kai Ren, Liyun Zhang, Chao Xue, Weixun Duan, Jincheng Liu, Ren Cong

**Affiliations:** 1 Xi’an Medical University, Xi’an, China; 2 Department of Cardiovascular Surgery, Xijing Hospital, The Air Force Medical University, Xi’an, 710032 Shaanxi China; 3 Department of Cardiovascular Surgery, The First Affiliated Hospital, The Air Force Medical University, Xi’an, 710032 Shaanxi China

**Keywords:** Aortic trauma, Blunt aortic injury, Traumatic aortic injury

## Abstract

**Background:**

Approximately 80% of patients with blunt thoracic aortic injury (BTAI) die before reaching the hospital. Most people who survive the initial injury eventually die without appropriate treatment. This study analyzed and reported the treatment strategy of a single center for BTAI in the last 10 years and the early and middle clinical results.

**Methods:**

This retrospective study included patients diagnosed with BTAI at Xijing Hospital from 2013 to 2022. All inpatients with BTAI aged ≥ 18 years were included in this study. The clinical data, imaging findings, and follow-up results were retrospectively collected and analyzed. The Kaplan–Meier curve and multivariate logistic regression were used to compare survivors and nonsurvivors.

**Results:**

A total of 72 patients (57% men) were diagnosed with BTAI, with a mean age of 54.2 ± 9.1 years. The injury severity score was 24.3 ± 18, with Grade I BTAI1 (1.4%), Grade II 17 (23.6%), Grade III 52 (72.2%), and Grade IV 2 (2.8%) aortic injuries. Traffic accidents were the main cause of BTAI in 32 patients (44.4%). Most patients had trauma, 37 had rib fractures (51.4%), Sixty patients (83.3%) underwent thoracic endovascular aortic repair (TEVAR) surgery, eight (11.1%) underwent conservative treatment, and only four (5.6%) underwent open surgery. The overall hospitalization mortality was 12.5%. In multivariate logistic regression, elevated creatinine levels (*P* = 0.041) and high Glasgow coma scale (GCS) score (*P* = 0.004) were the predictors of hospital mortality. The median follow-up period was 57 (28–87) months. During the follow-up period, all-cause mortality was 5.6% and no aortic-related deaths were reported. Three patients (4.2%) needed secondary surgery and two of them underwent endovascular repair.

**Conclusion:**

Although TEVAR surgery may be associated with intra- or postoperative dissection rupture or serious complications in the treatment of Grade III BTAI, the incidence rate was only 8.9%. Nevertheless, TEVAR surgery remains a safe and feasible approach for the treatment of Grade II or III BTAI, and surgical treatment should be considered first,. A high GCS score and elevated creatinine levels in the emergency department were closely associated with hospital mortality. Younger patients need long-term follow-up after TEVAR.

## Introduction

Trauma is still the most common cause of death in young people. Several autopsy reports have shown that head injury was the most common cause of death, followed by blunt thoracic aortic injury (BTAI) in patients with blunt trauma [1–3]. Blunt aortic injury is most commonly observed after a sudden deceleration, usually in a car accident [4]. Other causes include motorcycle, aircraft, car, and pedestrian collisions; falls; and crush injuries [5–8]. Approximately 80% of patients died before hospital arrival, and most of the deaths occurred within the first hour of hospital arrival [2, 3, 9].

The preliminary diagnosis of BTAI is mainly performed using computed tomography angiography (CTA), and subsequent imaging is performed using magnetic resonance angiography [10–12]. The Society of Vascular Surgery (SVS) classified BTAI as follows: Grade I, intimal tear; Grade II, intramural hematoma; Grade III, pseudoaneurysm; and Grade IV, free rupture. The common treatment methods are classified into three categories: conservative treatment, thoracic endovascular aortic repair (TEVAR), and open surgery. In 2011, the BTAI clinical practice guideline of the American SVS recommended that TEVAR can be considered superior to open repair or nonsurgical treatment, and in 2017, the European SVS recommended that TEVAR can be used as a first-line treatment [13]. Although the guidelines recommend surgical treatment (preferably TEVAR) for Grades II–IV, there is scarce evidence on the long-term prognosis of TEVAR. In this study, we attempted to review the past 10-year mortality rate of patients with BTAI in a single center to confirm the reliability and long-term efficacy of TEVAR, analyze the factors influencing death in hospitalized patients, and determine the trauma mechanism, trauma nature, and treatment results of Asian patients with BTAI.

## Methods

### Study design

This retrospective study was approved by the ethics committee of the First Affiliated Hospital of the Fourth Military Medical University (No. 20120216-4), and informed consent was obtained from patients. From July 2013 to March 2022, a total of 78 patients were diagnosed with BTAI. Their medical records were reviewed and analyzed. Based on electronic medical records and imaging data, six patients with aortic trauma occurring in the abdominal aorta and those aged < 18 years with penetrating injury were excluded. Finally, 72 patients with BTAI were included in this study. The baseline data, treatment details, and postoperative results of patients were retrospectively reviewed to obtain the following information: age, gender, trauma mechanism, trauma nature, related injuries, symptoms, vital signs and laboratory findings in the emergency department (ED), aortic injury score, injury severity score (ISS), treatment strategy (nonsurgical, open surgery, and endovascular repair), hospitalization events, and outcomes (hospitalization mortality, follow-up imaging, follow-up mortality, and follow-up secondary surgery). The diagnosis of BTAI is confirmed using computer tomography or CTA. If the patient’s condition is complicated, the surgical decision should be guided by transesophageal echocardiography to examine the presence of other cardiac malformations. This study lacks pre-hospital data and emergency information during the first visit of transferred patients, which may cause a deviation in the study population.

### Surgical procedure

Patients with BTAI underwent a femoral artery approach during TEVAR. The conventional operation was to cut through the right groin, dissociate a segment of the femoral artery, and perform femoral artery puncture and intubation. The 5F or 6F pigtail tube was subjected to angiography of the ascending aorta via the subclavian artery. The automatic contrast agent injection system was connected to the pigtail tube to perform aortic arch angiography. The size and location of the aortic aneurysm were observed, and the location of the rupture was re-determined. Transcatheter delivery of a guidewire to the ascending aorta. The catheter was then withdrawn, and the stent conveyor with membrane was placed. The stent was released under X-ray positioning. Follow-up CTA was performed at 1, 3, 9, and 12 months after treatment and annually thereafter to evaluate the position of stent implants, internal leakage, and aortic lesion changes. Survival was assessed by conducting outpatient visits or telephone interviews.

Open surgery for the left posterolateral approach is the preferred surgical approach, and all repairs are performed under cardiopulmonary bypass. Only sternotomy was performed for patients with a blunt injury involving the ascending aorta (conventional application of bentall surgery).

### Statistical analysis

All continuous variables were recorded as mean (± standard deviation) and medians (range), and categorical variables were expressed as percentages and numbers. Patients were classified as Grade II or Grade III BTAI based on hospitalization (survival and death) and TEVAR surgical outcomes. The independent sample *t*-test was used to analyze normally distributed continuous data, whereas Mann–Whitney *U*-test was used for non-normally distributed data. Pearson’s chi-square test and Fisher’s exact probability method were used to describe the classification of variables. The Kaplan–Meier curve was constructed to present the survival analysis of patients based on BTAI. A multivariate logistic regression model was used to calculate the propensity scores of survivors and nonsurvivors. The covariates included age, gender, ISS score, emergency GCS, emergency creatinine level, aortic injury type, and surgical method. A *P*-value of < 0.05 indicated statistically significant difference. Database management and statistical analysis were performed using the SPSS 25.0 software (IBM-SPSS Inc.).

## Results

### Patient characteristics

Patient characteristics (Table 1) included the baseline characteristics of 72 patients with BTAI with a mean age of 54.2 ± 8.9 years and comprised 57 (79.2%) men. Hypertension was the most common complication in 27 patients (37.5%), and 34 patients (47.2%) arrived at the hospital within 24 h after trauma. The Glasgow coma scale (GCS) score was 14.4 ± 1.7, and the ISS was 24.3 ± 18 on hospital arrival. Five patients (6.9%) had 120 mmHg systolic blood pressure per min on hospital arrival. Sixty patients (83.3%) experienced chest or back pain. The mechanism of injury and details of aortic injury are presented in Table 2. Traffic accidents were the most common injury mechanism in 32 patients (44.4%), followed by falling injuries and pedestrian–vehicle collisions. The most common site of aortic injury was the aortic isthmus (50%), followed by the aortic arch (25%). Most patients had Grade III (72.2%) and II (23.6%) aortic injury, and a few patients had Grade I (1.4%) and Grade IV (2.8%) injuries.

**Table 1:** Demographics and characteristics

Variable	Value
Number of all patients	72
Age, mean (SD), y	54.2 (9.1)
Male, n (%)	57 (79.2)
Hypertension, n (%)	27 (37.5)
Diabetes, n (%)	3 (4.2)
COPD, n (%)	0 (0)
Coronary heart disease, n (%)	1 (1.4)
Renal insufficiency, n (%)	2 (2.8)
Smoking, n (%)	30 (41.7)
Travel time to hospital, (median (IQR), days)	2 (1–6)
Travel time to hospital ≤ 24 h	34 (47.2)
Hypotension (SBP < 90) at ED, n (%)	5 (6.9)
Heart rate at ED > 120, n (%)	9 (12.5)
GCS at ED, mean (SD)	14.4 (1.7)
ISS score at ED, mean (SD)	24.3 (18)
Creatinine at ED, mean (SD)	95.9 (95)
Oximetry at ED, mean (SD)	95.6 (5.2)
prothrombin time at ED, mean (SD)	15.6 (23.3)
Anterior or posterior chest pain, n (%)	60 (83.3)
Intubation, n (%)	1 (1.4)

SD, standard deviation; IQR, interquartile range; COPD, chronic obstructive pulmonary disease; GCS, glasgow coma scale; ISS, injury severity score

**Table 2:** Mechanism of injury and details of aortic injury

Variable	Value
Causes of injury, n (%)	
Car accidents	32 (44.4)
Auto-pedestrian collisions	11 (15.2)
Motorcycle accidents	3 (4.1)
Fall < 3 m	8 (11.1)
Fall ≥ 3 m	12 (16.7)
Falling object	1 (1.4)
Others	5 (6.9)
Concomitant injuries, n (%)	
Fractured ribs	37 (51.4)
Haemothorax	26 (36.1)
Pneumothorax	3 (4.2)
Abdominal injury	10 (13.9)
Pelvic injury	18 (25)
Traumatic brain injury	23 (31.9)
Mean chest AIS at ED, mean(SD)	3.7 (1.2)
Mean head AIS at ED, mean(SD)	3.5 (1.4)
Location of aortic injury, n (%)	
Ascending aorta	9 (12.5)
Aortic arch	17 (23.6)
Isthmus	37 (51.4)
Descending aorta	9 (12.5)
Grade of aortic injury, n (%)	
I	1 (1.4)
II	17 (23.6)
III	52 (72.2)
IV	2 (2.8)
Admission EF (%), mean(SD)	56.9 (4.8)
Annulus diameter, mean (mm), mean (SD)	22.6 (2.2)
SOV (mm), mean (SD)	32.7 (4.9)
Ascending aortic diameter (mm), mean (SD)	31.3 (3.1)

AIS, abbreviated injury score; ED, emergency department; SOV, sinuses of Valsalva

### Treatment methods and results

The treatment methods and results are shown in Table 3. Twenty-nine patients (40.2%) underwent emergency surgery, 35 (48.6%) underwent delayed surgery, and eight (11.1%) did not undergo surgery. Among the 8 patients (11.1%), 1 patient with Garde IV died during preoperative transport due to dissection rupture and heart failure, and 2 patients with Garde III died during hospitalization. One of them had a history of cerebral infarction and the other had multiple organ failure and severe craniocerebral injury. Both failed to meet the surgical conditions. Among the five patients who continued to receive conservative treatment after discharge, one Garde III patient was converted to conservative treatment due to the absorption of hematoma during the preparation of thoracotomy due to dissection in the ascending aorta. One Garde III patient failed to meet the surgical indications due to multiple organ failure and heart failure. One Garde III patient refused surgery for economic reasons. The other two Garde I and Garde II patients had mild injury. All five patients were followed up so far without serious complications and postoperative death. Among the 60 patients (83.3%) receiving TEAVR surgery, One case died of hemorrhagic shock due to ruptured dissection after stent release. One patient developed systemic inflammatory response syndrome after the surgery and died due to a massive blood inflow of inflammatory factors. One patient refused further treatment due to preoperative cerebral infarction, pelvic fracture, and severe liver dysfunction. The family members of patients with postoperative neurological dysfunction also refused further treatment. One patient did not undergo treatment due to postoperative liver dysfunction and subarachnoid hemorrhage. Of the four patients (5.6%) who underwent open surgery, two underwent Stanford type A aortic dissection. One patient died due to intraoperative dissection rupture and hemorrhagic shock, and another patient died due to excessive postoperative bleeding. The all-cause mortality rate was 12.5%, and the aortic-related mortality rate was 4.2%. The study ended on May 16, 2022. A total of 63 patients who survived and were discharged from the hospital were followed up successfully. The median follow-up time was 57 (28–87) months. Four patients died during this period. No serious postoperative complications or death occurred during the first year of follow-up, and the short-term outcome was excellent. One patient committed suicide due to mental illness 2 years after the surgery. Another patient died of hemorrhagic shock due to massive gastrointestinal bleeding 2 years after the surgery. One patient died after secondary trauma 5 years after the surgery. Finally, the last patient died of liver cancer 7 years after the surgery. No deaths due to aortic complications were recorded. During the follow-up period, three patients had the following secondary cardiovascular surgery records: the placement of stents for the heart due to myocardial infarction, secondary placement of stents for luminal stenosis, and replacement of the aortic valve. 1 case received secondary stent placement 5 years after operation, which was not related to the first lesion, and 1 case underwent aortic valve replacement in our hospital 8 years after operation due to aortic valve insufficiency. About the stent graft, we only saved the data of admission after 16 years. We analyzed and counted the 40 patients (40/60) and found that the diameter of the stent graft anchoring area was mostly 28 mm (median: 28 IQR: 26–28). Among them, 20 patients were placed in two stents by overlapping three sections. The diameter and length of the stent graft were about 28 mm (median: 28; iQR: 28–30); 150 mm (median: 150; iQR: 120–150).

**Table 3:** Procedural details and post-operative outcomes

Variable	Value
Nonoperative, n (%)	8 (11.1)
Open surgery, n (%)	4 (5.6)
Endovascular stent, n (%)	60 (83.3)
Preoperative death after admission, n (%)	1 (1.4)
Rupture in operative room, n (%)	2 (2.8)
Emergency surgery, n (%)	29 (40.2)
Delayed surgery, n (%)	35 (48.6)
Total ventilation days (median (IQR), days)	1 (1–3.6)
Reintubation, n (%)	4 (5.6)
Postoperative admission to ICU, n (%)	14 (19.4)
Postoperative ICU time (mean ± SD, hours)	49.4 (39.4)
Paraplegia, n (%)	1 (1.4)
Pulmonary embolism, n (%)	1 (1.4)
Irenal failure, n (%)	1 (1.4)
Length of hospital stay (mean ± SD, days)	8.3 (7.7)
In-hospital mortality, n (%)	9 (12.5)
Duration of follow-up (median (IQR), months)	57 (28–87)
Mortality during follow-up, n (%)	4 (5.6)
Mortality during follow-up related to aortic cause, n (%)	0 (0)
Secondary surgery during follow-up, n (%)	3 (4.2)
Secondary surgery related to aortic cause, n (%)	2 (2.8)
Replacement of valves, n (%)	1 (1.4)

ICU, intensive care unit

Table 4 compares the effects of TEVAR on Grade II and III aortic injuries. Although Grade III aortic injury shows a higher probability of postoperative intensive care unit (ICU) admission and hospital mortality, no significant difference was observed between the two groups. Table 5 compares the characteristics of survivors and nonsurvivors. No difference was observed in factors, such as age, sex, and hypertension. Nonsurvivors had lower GCS scores (*P* = 0.001), elevated creatinine levels (*P* = 0.001), greater number of brain injuries (*P* = 0.045), higher aortic injury grades (*P* = 0.004), and lower proportion of endovascular treatment (*P* = 0.013).

**Table 4:** Comparison of clinical features and results between grade II and grade III patients with endovascular stent

Characteristics	Grade2 (n = 15)	Grade3 (n = 45)	*p* value
Age, mean (SD), y	50 (10.1)	49.5 (14.9)	0.77
Male, n (%)	11 (18.3)	35 (58.3)	0.73
Hypertension, n (%)	4 (6.7)	18 (30)	0.35
Travel time to hospital ≤ 24 h	8 (13.3)	21 (35)	0.66
GCS at ED, mean (SD)	15 (0)	14.4 (1.7)	0.026
ISS score at ED, mean (SD)	23.1 (16.1)	26 (17.7)	0.57
Causes of injury, n (%)			0.535 for all
Car accidents	9 (15)	19 (31.7)	
Auto-pedestrian collisions	1 (1.7)	9 (15)	
Motorcycle accidents	1 (1.7)	1 (1.7)	
Fall < 3 m	1 (1.7)	4 (6.7)	
Fall ≥ 3 m	2 (3.3)	9 (15)	
Falling object	0 (0)	1 (1.7)	
Others	1 (1.7)	2 (3.3)	
Concomitant injuries, n (%)			
Fractured ribs	11 (18.3)	20 (33.3)	0.053
Haemothorax	5 (8.3)	16 (26.7)	0.87
Pneumothorax	1 (1.7)	2 (3.3)	0.74
Abdominal injury	2 (3.3)	7 (11.7)	0.83
Pelvic injury	2 (3.3)	14 (23.3)	0.31
Traumatic brain injury	2 (3.3)	21 (35)	0.021
Location of aortic injury, n (%)			0.26 for all
Ascending aorta	1 (1.7)	2 (3.3)	
Aortic arch	6 (10)	7 (11.7)	
Isthmus	6 (10)	29 (48.3)	
Descending aorta	2 (3.3)	7 (11.7)	
Postoperative admission to ICU, n (%)	0 (0)	8 (13.3)	0.18
In-hospital mortality, n (%)	0 (0)	4 (6.7)	0.56
Mortality during follow-up, n (%)	1 (1.7)	3 (5)	1
Secondary surgery during follow-up, n (%)	1 (1.7)	2 (3.3)	0.74

**Table 5:** Comparison of clinical features and management methods between survivors and nonsurvivors

	Survivors (n = 63)	Fatalities (n = 9)	*P* value
Age, mean (SD), y	54.2 (9.1)	48.9 (12.4)	0.98
Male, n (%)	48 (66.7)	9 (12.5)	0.19
Hypertension, n (%)	23 (31.9)	4 (5.6)	0.927
Travel time to hospital ≤ 24 h	30 (41.7)	4 (5.6)	0.92
GCS at ED, mean (SD)	14.8 (0.9)	10.8 (2.3)	0.001
ISS score at ED, mean (SD)	24 (18.2)	34.8 (10.3)	0.18
Creatinine, mean (SD)	75.3 (25.5)	209 (242.5)	0.001
Prothrombin time, mean (SD)	15.3 (25.5)	17.7 (8.9)	0.63
Fractured ribs, n (%)	33 (45.8)	4 (5.6)	0.73
Abdominal injury, n (%)	2 (2.8)	1 (1.4)	0.33
Pelvic injury, n (%)	16 (22.2)	2 (2.8)	0.83
Traumatic brain injury, n (%)	17 (23.6)	6 (8.3)	0.045
Location of aortic injury, n (%)			0.11for all
Ascending aorta	6 (8.3)	3 (4.2)	
Aortic arch	15 (20.8)	3 (4.2)	
Isthmus	33 (45.8)	3 (4.2)	
Descending aorta	9 (12.5)	0 (0)	
Grade of aortic injury, n (%)			0.007for all
I	1 (1.4)	0 (0)	
II	17 (23.6)	0 (0)	
III	45 (62.5)	7 (9.7)	
IV	0 (0)	2 (2.8)	
Length of hospital stay (mean ± SD, days)	8.2 (6.3)	7.9 (6.2)	0.875
Nonoperative, n (%)	5	3	0.013 for all
Open surgery, n (%)	2	2	
Endovascular stent, n (%)	56	4	

**Fig. 1:**
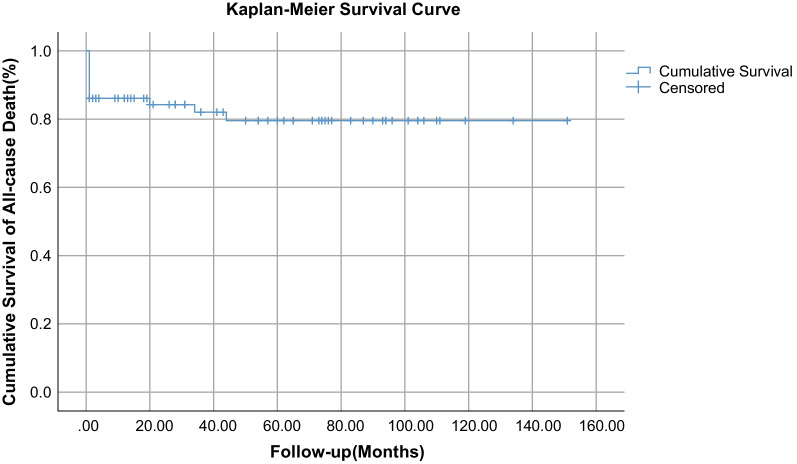
Curves of cumulative survival of all-cause death

The overall survival of all patients was described in the Kaplan–Meier curve in Fig. 1. The overall five-year projected survival rate is about 80%. In addition, multivariate logistic regression analysis showed that the GCS scores in the ED (odds ratio [OR] 0.374; 95% confidence interval [CI] 0.192–0.727; *P* = 0.004) and creatinine levels in the ED (OR 1.037; 95% CI 1.001–1.074; *P* = 0.041) were statistically significantly associated with death events during hospitalization (Table 6).

**Table 6:** Multivariate logistic regression analysis on mortality of BTAI patients

Variables	Odds ratio	95% CI		*P* value
		Lower	Upper	
ISS	1.069	0.971	1.175	0.173
Creatinine	1.037	1.001	1.074	0.041
GCS	0.374	0.192	0.727	0.004

## Discussion

This study reported data on a group of patients with BTAI who visited Xijing Hospital during the last 10 years. The retrospective study data were extracted from hospital records based on the Chinese aortic dissection registry database, supplemented by the hospital cardiosurgery database and electronic medical records.

In our study, the male population was predominant (79.2%), with hypertension (37.5%) as the most common complication, traffic accidents as the most common injury mechanism (44.4%), and aortic isthmus (51.4) as the most common injury site. These basic patient characteristics were consistent with those of patients in other centers. However, compared with those in France and the United States, the age of patients in China in this study was significantly older than 54.2 ± 9.1 years [8, 14–17].

This study applies the classic grading system [11] of the SVS. Compared with single-center studies in other countries [5, 8], Grade III lesions accounted for a higher proportion of injuries in this study, which may be the most common type of BTAI in China. Further, this is closely associated with the following two facts (1). Although chest or back pain were found in 83.3% of patients in this study, the causes of pain are complex and diverse, and rib fractures, spinal injuries, pelvic fractures, and chest soft tissue injuries are prone to missed diagnosis or misdiagnosis [10, 18]. The main reason for the missed diagnosis was that orthopedic experts and ICU bedside doctors did not thoroughly examine the thoracic aorta injury and did not conduct aortic CTA examination or cross-sectional imaging to investigate vascular injury when the patient first visited the ED [10, 11, 19, 20]. In this study, 13 patients (18%) were treated in the local lower-level hospital for several weeks and months after trauma; however, symptoms did not show any improvement and no treatment required referral to cardiovascular surgery in our hospital. Emergency surgeons must understand the correlation between blunt thoracic trauma and BTAI, and based on the injury mechanism of patients with trauma, BTAI should be highly suspected. (2) Approximately 80% of patients with blunt aortic injury die before reaching the hospital, and most of those who survive the initial injury die without appropriate treatment [1, 2, 11, 18, 21, 22], which may result in a deviation in the analysis population.

Based on the guidelines, Grade II–IV injuries should be treated via surgery. In this study, TEVAR was given priority for the treatment of patients with Grade II and III thoracic aortic injuries. Several previous studies have reported the beneficial results of endovascular repair on aortic trauma. Thomas M Scalea et al. [9] used the American College of Surgeons National Trauma Databank (2003–2013) to identify adults with BTAI and found that TEVAR largely replaced open surgery, thereby reducing the mortality rate of BTAI by 50%. In both meta-analyses [23, 24], endovascular intervention was associated with a significantly lower paraplegia rate and a lower mortality rate in traumatic aortic injury compared with open surgery. Endovascular intervention due to the avoidance of aortic clamping reduced intraoperative blood loss and did not require the use of postoperative anticoagulants, resulting in an increase in the use of this technology every year [9, 25, 26]. Thus, open surgery and endovascular intervention in this study were not indicated for postoperative patients with paraplegia, and an effective conclusion cannot be drawn. A recent study published by Alexey Kamenskiy et al. [27] reported that TEVAR in patients with BTAI may lead to accelerated expansion and reconstruction of the ascending aorta, increased left ventricular mass, and high incidence of hypertension. Therefore, long-term prevention of cardiovascular complications and follow-up or even lifelong detection for young patients should be implemented after the surgery. Al-Thani et al. [26] reported that GCS and aortic injury grade in the ED were independent predictors of mortality in patients with BTAI. The 5-year survival rate after TEVAR was 94%, and the 5- and 10-year survival rates reported by Agostinelli et al*.* [7] were 92% and 87%, respectively. The 5- and 10-year survival rates of TEVAR in this study were 97% and 84%, respectively, which were similar to the results of the two studies. The mean time required to perform TEVAR in this study was 41 ± 14.9 min, which was significantly shorter than that in other centers (80.5 ± 59.9 min) [28]. No study has confirmed the difference between the time required to perform TEVAR and postoperative results; therefore, further studies are warranted in the future.

Recent studies have shown that the nonsurgical treatment of patients with low-grade (Grade I–II) BTAI does not lead to long-term aortic complications or require further intervention. Nonsurgical treatment can be safely used for Grade II BTAI [29, 30]. We need to understand which patients in conservative treatment can better absorb hematoma, which may require further basic research to support. In the conservatively treatment group, the indication for surgery was not met due to extremely poor cardiopulmonary function or organ failure, and 5 of 8 deaths (62.5%) were reported due to the refusal of surgery by family members. Three patients died during hospitalization, and only two patients were followed up. The management of patients who opted for nonsurgical treatment also needs urgent relevant research and analysis. Overall, different surgical management strategies and inconsistent intensive care programs may lead to different mortality rates.

## Conclusion

TEVAR surgery is safe and feasible for patients with BTAI with Grade II–III aortic injury. Due to the lack of long-term imaging follow-up, long-term multicenter follow-up is needed to evaluate the long-term efficacy of TEVAR in younger patients in the future. In our study, the total mortality of patients with BTAI was correlated with high GCS scores and creatinine levels.

## Data Availability

The datasets generated and/or analyzed during the current study are not publicly available due to the use of internal records of patient data and the established privacy policy but are available from the corresponding author upon reasonable request.

## Abbreviations

ED: Emergency department
SD: Standard deviation
IQR: Interquartile range
COPD: Chronic obstructive pulmonary disease
GCS: Glasgow coma scale
ISS: Injury severity score
AIS: Abbreviated injury score
SOV: Sinuses of Valsalva
ICU: Intensive care unit
BTAI: Blunt thoracic aortic injury
